# Assessment of practical science in high stakes examinations: a qualitative analysis of high performing English-speaking countries

**DOI:** 10.1080/09500693.2020.1769876

**Published:** 2020-08-06

**Authors:** Sibel Erduran, Yasmine El Masri, Alison Cullinane, YPD Ng

**Affiliations:** a Department of Education, University of Oxford, Oxford, UK; b The Norwegian Centre for Science Education, University of Oslo, Oslo, Norway

**Keywords:** Assessment, PISA, examinations, high-stakes testing

## Abstract

High stakes examinations can have profound implications for how science is taught and learned. Limitations of school science such as the ‘cookbook problem’ can potentially be addressed if high stakes assessments target learning outcomes that are innovative. For example, less mindless procedural engagement and more thoughtful consideration of practical science can potentially improve science learning. In this paper, we investigate how practical work is represented in the assessment frameworks of several countries that demonstrate above average performance in the latest PISA science assessments. The main motivation is the need to understand if there are aspects of high stakes summative assessments in these countries that can provide insight into how best to structure national examinations. Assessment documents from a set of selected countries have been analysed qualitatively guided by questions such as ‘what is the construct of practical science’ and ‘what is the format of assessment?’ The examined jurisdictions used different approaches from traditional external pen-and-paper tests to internal teacher assessments that included different formats (e.g. laboratory report). Innovative approaches to the assessment of practical skills (e.g. PISA computer-based tasks) do not seem to be represented in these high-stakes assessments. Implications for innovative assessments for high-stakes purposes are discussed.

## Introduction

Practical work has been a component of the science curriculum in many parts of the world for a long time (Bybee, 1997; Donnelly, 1994). Research in science education (Abrahams et al., 2013) points to the key role that assessments play in shaping the curriculum content of practical work. There is widespread recognition that summative assessment drives what is taught in science lessons (Abrahams & Saglam, 2010). In examining what is typically taught with respect to practical science exposes that students are engaged in procedures that do not make sense from their points of view. Mindless pursuit of procedures has typically been referred to as the ‘cookbook problem’. As Leonard (1991) states, practical work in science ‘is very prescribed, that is, the student is told what to do in a step-by-step fashion for the entire exercise’. (p. 84)

Students in science lessons understandably question why they are doing what they are doing. They ask such questions because while the purpose of an investigation may be apparent to the teachers, it may not be so obvious to the students. For example, students may not understand why they have to take a measurement such as the volume of a liquid when this measurement is so far removed from building an explanation about a phenomenon such as titration. It may be implicit from the teacher’s perspective that the volume will lead to a calculation of the concentration of an acid. However, from the student’s point of view, the procedure of measurement is simply an action that is fairly removed from the abstract explanations that will subsequently be generated using the data. Some students may recognise the significance of measurement upfront but many do not actually understand why they are doing what they are doing in practical work in science lessons. Thus, researchers have been noting for decades that students need to engage in practical activities that have a closely aligned explanatory framework to give them a sense of purpose in their learning (Bransford et al., 1999).

An aspect of recipe following in practical work involves hypothesis testing in experiments. Students are indoctrinated into a perspective of science as hypothesis testing, with associated concepts of dependent and independent variables taught without much thinking devoted to how different questions might demand other scientific methods (Erduran & Dagher, 2014). Knowing the importance of gathering empirical evidence in science is a good starting point, but addressing why and how this evidence is collected, what methodological rules guide its collection, and how reasoning tools function in validating and justifying their collection cannot be separated from the procedures of data collection and interpretation.

How practical science is assessed in high stakes examinations can have profound implications for how it is taught in science lessons such that limitations of school science such as the ‘cookbook problem’ can be surmounted. In this paper, we investigate how practical science is represented in the assessment frameworks of several English-speaking countries that demonstrate above average performance in the latest PISA science assessments. As the rest of the paper will demonstrate, there is a range of terminology related to practical work in science. For the sake of simplicity, we will refer to our notion of practical work as ‘practical science’. We define ‘practical science’ as an overarching term that refers to any type of science teaching and learning activity in which students are involved in manipulating and/or observing objects and materials in order to understand how a particular phenomenon works.

The overall question guiding the research is ‘What is the nature of assessment frameworks in relation to practical science in a sample of English-speaking countries that performed higher than the OECD average in the 2015 PISA science test?’ The main motivation underlying the question is to identify aspects of high-stakes summative assessments in these countries that could have contributed to their high performance in science. Understanding such aspects of assessments can potentially provide insight into how best to structure national examinations elsewhere. The reference to ‘English-speaking’ is a pragmatic issue primarily due to the limitations of the authors in accessing documents published in other languages. In order to address the main question, we performed a qualitative analysis guided by a set of questions about the nature of practical science and assessment represented in the documents. The main inclusion criterion focused on the countries performing well in PISA science 2015 (i.e. national mean score in science is higher than the OECD average).

Qualitative analysis (Nowell et al., 2017) was conducted to investigate particular aspects of summative assessments including (a) construct of practical science, (b) format of assessment, (c) marking procedures and (d) mechanisms of quality control. The overall aim is to examine to what extent there may be innovation in assessment of practical science in these documents. Innovation is defined in relation to a set of examples from existing summative assessments that are not part of high-stakes examinations. For example, it can include computer-based tasks (e.g. PISA science) and scenario analysis (e.g. business administration). Features of such assessments will be reviewed along with a broad overview of literature background on high-stakes assessment of practical science.

## Literature review

### High-stakes assessment of practical science

There is different terminology related to ‘practical science’ which can be confusing in setting assessment objectives. For example, the terms used include *experimental work, practical and enquiry skills*, *practical and investigative activities* (Qualifications and Curriculum Authority, 2007), *practical experimentation,* and *practical scientific methods* (Department for Education, 2014). The ambiguity in the language around practical science is compounded with an overemphasis on the experimental method even though scientists also use other methods such as non-manipulative observations and manipulative descriptions (Erduran & Dagher, 2014). When the diversity of methods is overlooked in science education, knowledge obtained from non-experimental methods, such as non-manipulative descriptive classification (e.g. classification of elements in the Periodic Table), is likely to be viewed by teachers and pupils as less privileged or less important than rigorous hypothesis testing (e.g. investigating the effect of temperature on the pressure of a gas) (Erduran & Dagher, 2014).

The objectives of doing practical science include motivation for students, consolidation of theory, development of manipulative skills and understanding of data handling. Conventionally, practical science has been advocated due to the promotion of process skills, assuming that these skills are ‘generalisable, transferable from one context to another and readily applicable in any context’ (Hodson, 1994, p. 159). Although practical skills include students’ competence about the manipulation of equipment and apparatus, there are a large number of such skills, making it unfeasible to assess all of them within the limited time available in school science (Reiss et al., 2012). Furthermore, different employers may have very different perspectives on which practical skills they consider important. Despite the development of a range of practical skills in school science, there is still evidence that graduates are not equipped with appropriate skills for the workplace. For example, the Confederation of British Industry (2011) reported that 23% of employers felt that the lack of practical experience and laboratory skills was a barrier to recruitment of STEM-skilled staff.

Despite extensive emphasis on practical science in school science and on assessment more generally, the literature on the assessment of practical science in science education is limited (Abrahams et al., 2013). Some authors have also pointed out that specifications in the summative assessments may not be helpful in promoting meaningful engagement in practical science. For example, Wilson (2013) states:

However, there has been some serious questioning of the efficacy of work done in science practical lessons. There is also a consensus that the type of isolated practical tasks carried out for assessment purposes at GCSE do not enable students to find out how scientific enquiry really works. As such, the forms of summative assessment currently used at GCSE are considered not to support the aims of practical science. (p. 4)

Furthermore, there are issues with the validity of assessments related to practical science. There may be concerns about their reliability. For example, in a report on the testing of practical skills in science for ages 11, 13 and 15, Welford et al. (1985) suggested, that ‘the assessment of practical skills may be possible from pupils’ reports or write-ups – provided that they have actually carried out the practical or investigation prior to putting pen to paper’ (p. 51). Reiss et al. (2012) make a useful distinction can be made between what we refer to as direct assessment of practical skills (DAPS) and indirect assessment of skills (IAPS). The former, DAPS, refers to any form of assessment that requires students, through the manipulation of real objects, to directly demonstrate a specific or generic skill in a manner that can be used to determine their level of competence in that skill. An example of this would be if a student was assessed on their skill in using an ammeter and this was determined by requiring them to manipulate a real ammeter and use it within a circuit to take readings and for these readings need to be within an acceptable range of values for the student to be credited.

In contrast, IAPS relates to any form of assessment in which a student’s level of competency, again in terms of a specific or generic skill, is inferred from their data and/or reports of the practical work that they undertook. For example, when a student writes up an account of the reaction between hydrochloric acid and calcium carbonate chips in a way that the marker would not be certain if the student is writing what they have just done or simply remembering what they have previously done or been told about this reaction (Reiss et al., 2012).

Despite extensive emphasis on practical work in school science and on assessment more generally, the literature on the assessment of practical work in science education is still fairly limited (Reiss et al., 2012). First, as already stated, the different terminology related to ‘practical science’ can be confusing in setting assessment objectives. Some examination boards advocate that skills underpinning practical science should be assessed in a coordinated fashion. For instance, the Assessment and Qualifications Alliance (AQA) in England takes the position that these skills are separate but ‘interconnected’ (AQA, 2016, p. 2). Often however, curriculum innovation is often not coupled with the design of novel assessments (Linn et al., 1991) and/or accompanied by teacher engagement to ensure effective implementation of ambitious curricular goals (Ryder et al., 2014).

Further research and development on summative assessments is needed to investigate how practical science is related to (a) higher order thinking skills such as argumentation that involve skills such as evaluating evidence and justification of claims emerging from practical work, (b) procedural skills such as reliable measurement and observation using appropriate apparatus and techniques, and (c) knowledge of scientific vocabulary, quantities, symbols and nomenclature, culminating in meaningful attainment of skills required by the ‘Working Scientifically’ component of the Key Stage 4 curriculum (age 15–16) in England (DfE, 2014). The Office of Qualifications and Examinations Regulation (Ofqual) in England advocates that students ‘develop their ability to evaluate claims based on science through critical analysis of the methodology, evidence and conclusions, both qualitatively and quantitatively’ (Ofqual, 2015, p. 5). This point seems to suggest that the items specified above need to be taught and assessed in unison because the hands-on procedural skills embedded in methodology are coupled with minds-on approaches including critical analysis and evaluation.

The development of robust summative assessments is particularly critical given the widespread concern that students start higher education without sufficient skills in relating data collected through practical activities to theoretical knowledge that they gained from lectures, lacking coherence in their understanding of science (e.g. Andrews, 2010). Thus, it is important to understand how secondary students’ skills and knowledge of practical science can be measured not only in terms of the constituent aspects of practical science but also the coherent and holistic interpretation of these aspects together. However, such complex goals of assessment suggest a demand for innovation. Traditional forms of assessment such as pen-and-paper assessments may not be suitable. What, then, counts as innovation in summative assessment? In the next section, we address this question and review some examples.

### Innovation in summative assessment

Innovation in assessment of practical science refers to types of tasks that are different from the common pen-and-paper assessments that target knowledge of practical science (e.g. naming a product in a chemical reaction or describing certain procedures in a manipulation). These could form the basis of a more creative model for assessing practical science. In this section, examine (a) PISA science tasks (pen-and-paper and computer-based), (b) scenario analysis, and (3) objective structured clinical examinations (OSCEs) as examples of recent innovations in summative assessment.

### PISA test questions

**Figure 1.**
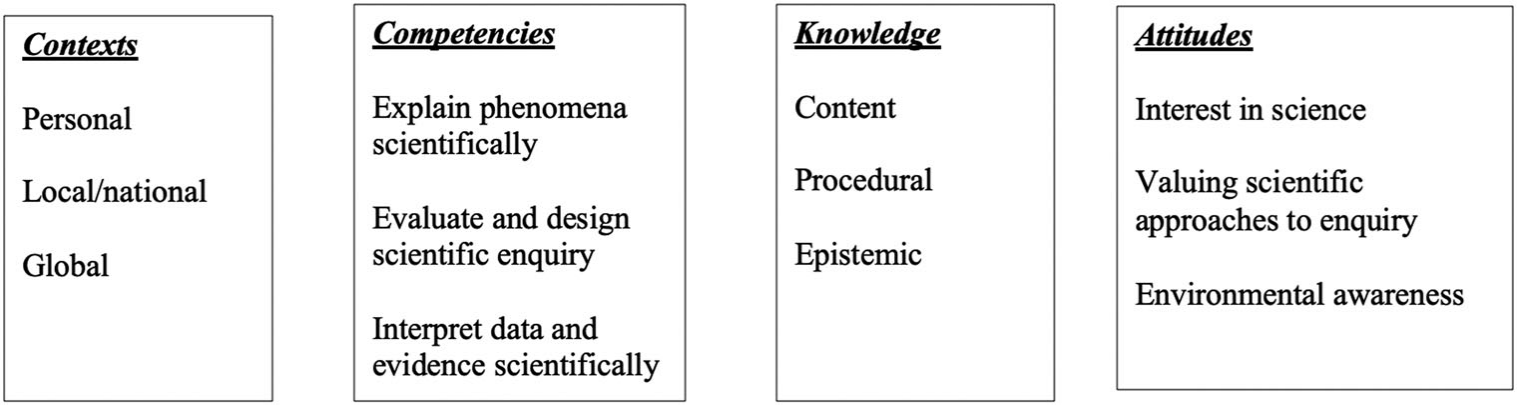
Science sub-domains in PISA 2015 (OECD, 2018).

PISA has been administered every 3 years to 15-year-olds by the Organisation for Economic Cooperation and Development (OECD) since 2000 and is claimed to be curriculum-independent as it evaluates what students can do using the knowledge and skills they have acquired by the end of compulsory education (OECD, 2016a). Among other subjects, the survey assesses students’ science literacy, that the OECD defines as ‘the ability to engage with science-related issues, and with the ideas of science, as a reflective citizen’ (OECD, 2016b, p. 20). Science literacy underlies most science education curricula around the world and has been adopted in England in the national science education curriculum since 2003 ‘Twenty-First Century Science’ (Bybee, 1997; DeBoer, 2000; Millar & Osborne, 1998). PISA science assessments are developed based on a framework. The latest version underlying the 2015 PISA science assessments comprises three inter-related sub-domains: Competency, Knowledge and System (see Figure 1).

**Figure 2.**
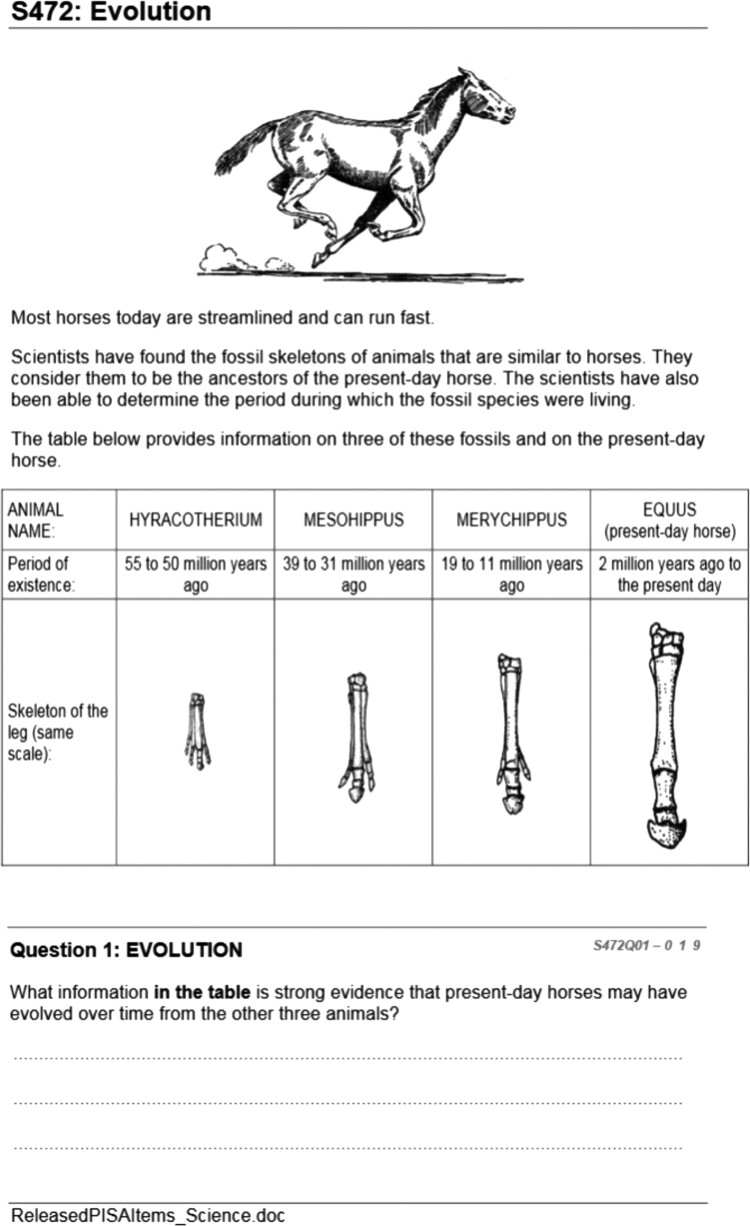
PISA released science item [S472 – Evolution].

Figure 1 illustrates how a range of practical science skills are captured in the assessment item. ‘Designing enquiries, interpreting data and evidence scientifically’ involves not only hands-on (i.e. designing enquiries) but also minds-on (i.e. interpreting data) skills. Given PISA’s focus on twenty-first century skills, including science investigative skills, we examine closely two types of PISA tasks: (a) tasks delivered as pen-and-paper tasks (pre-2015) and (b) interactive on-screen tasks (post-2015). PISA tasks in all subjects including science are organised in units consisting of stimulus material (i.e. text, photo, diagram, etc.) followed by a series of related questions (see example in Figure 2).

**Figure 3.**
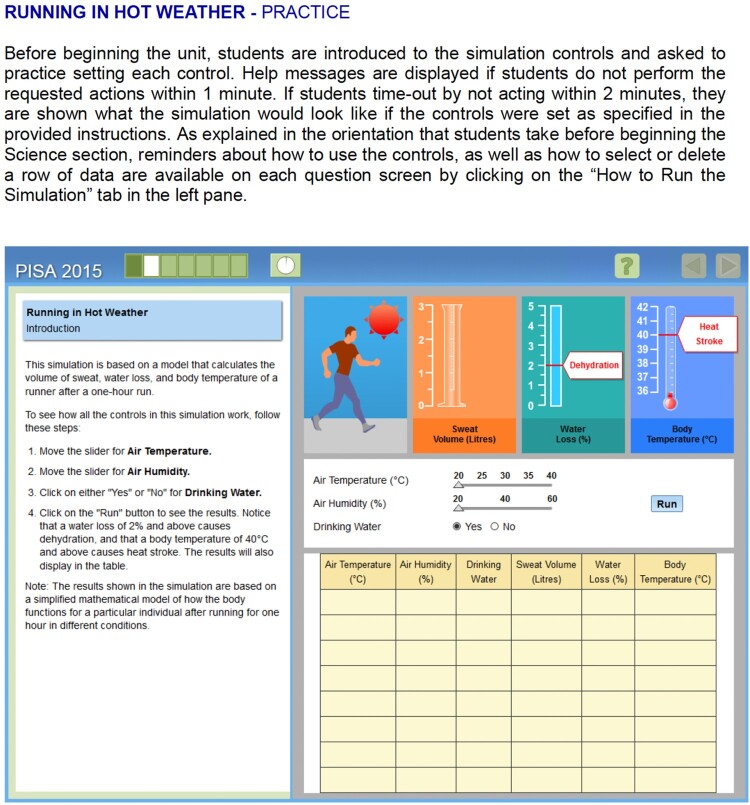
PISA computer-based science item (OECD, 2018).

The item in Figure 2 tests students’ observational skills and their ability to provide evidence supporting a hypothesis (e.g. evolution of present-day horses from three other animals). Since 2015, PISA has moved from pen-and-paper to computer-based assessments. Released examples of such interactive tasks can be found by visiting the following link: http://www.oecd.org/pisa/test Figure 3 provides one example to illustrate how technology can be incorporated in science summative assessments. The example shows an interactive task where candidates can vary some variables (temperature and humidity) and run simulations to answer specific questions. Tasks are preceded by an ‘Introduction’ describing a particular real-life scientific context (in this case, running in hot weather) and another ‘Practice’ page where students can click on various buttons to practice the simulation controls. The practice phase serves to limit the effect of low ICT literacy on performance on such items.

### Scenario analysis

Scenario analysis is described as an instructional tool that is used to investigate complex problems that sometimes can require trans-disciplinary knowledge and inquiry. The approach used in an MBA course described by Bradfield et al. (2015) states how it introduces the concept of ‘reframing’ narrowly defined issues in order to place them in a broader societal, economic and ecological context. This approach first affords the students to undertake semi-structured investigations into a particular issue and gives students the opportunity to steer the direction of the narratives of their learning. Students are required to present their developed scenarios, supporting research and also a critical reflection of their learning. Substantive knowledge and what they observed about the process and their own learning is captured in this reflective process. An example of scenario or focal issues used in the MBA course is as follows: *How – and to what extent – will current levels of poverty, infant mortality and educational provision change within a particular country over the next fifteen years?* (Bradfield et al., 2015).

While such examples are seemingly irrelevant for science lessons, the principles of the inclusion of transdisciplinary knowledge and inquiry can be integrated into the content of assessment in practical science. Consider, for example, the interplay of the topic of gene editing and economics. Erduran and Mugaloglu (2013) refer to the case of competing claims about a genetically modiﬁed mouse that was produced at Harvard University. The onco-mouse is a patented organism. It was designed to be susceptible to cancer and it is intended to help scientists understand cancer. The scenario of the onco-mouse lends itself to the surrounding economic as well as ethical issues that situate science as a social enterprise. Is it ethical to patent and sell an organism? Should access to scientiﬁc knowledge be restricted on economic grounds? The topic of DNA extraction lies at the heart of this scenario and is included in existing assessments (e.g. Pearson EdExcel, 2016). The scenario of practical DNA extraction lends itself to broader engagement with scenario analysis where related economic and societal issues can be examined.

### Objective structured clinical examinations (OSCEs)

Objective Structured Clinical Examinations (OSCEs) were first introduced in 1975 (Harden & Gleeson, 1979) and are described as a versatile tool that is multi-purposeful for evaluating and assessing health care professionals in clinical settings. These too are very similar to scenario analysis. Zayyan (2011) states that an OSCE

assesses competency, based on objective testing through direct observation. It is precise, objective, and reproducible allowing uniform testing of students for a wide range of clinical skills. Unlike the traditional clinical exam, the OSCE could evaluate areas most critical to performance of health care professionals such as communication skills and ability to handle unpredictable patient behaviour. (p. 219)

**Figure 4.**
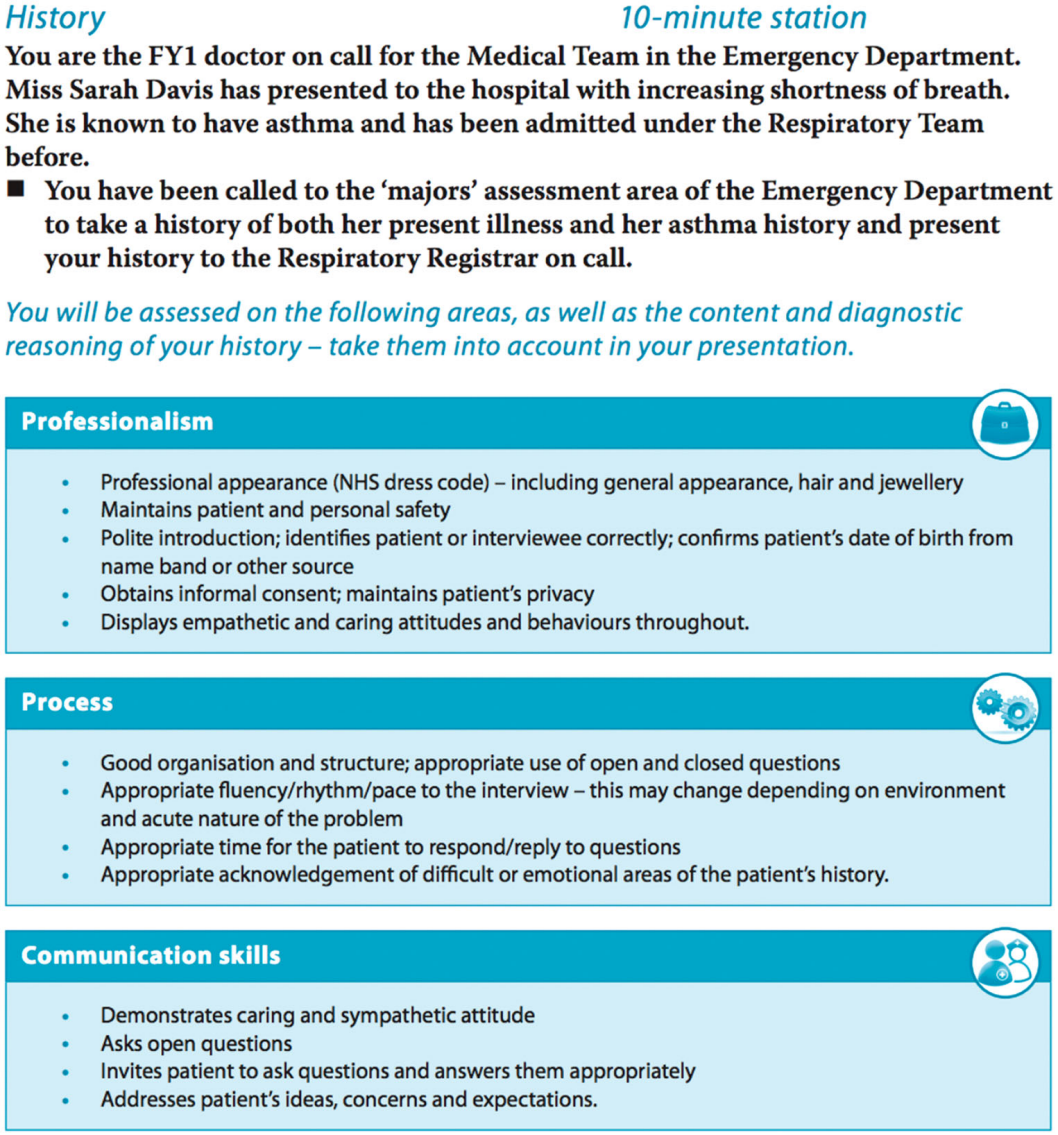
Example of an OSCE task (from Feather et al., 2012).

A typical OSCE comprises of a number of stations, where students have to use the information provided and analyse a given problem to make a diagnosis. Figure 4 below presents an example of scenario analysis from an OSCE and illustrates how tasks are framed. While OSCEs have been primarily developed for assessing clinical skills, they have been adapted to other fields of study (Bogo et al., 2014) and therefore could be adopted for assessing practical skills (Table 1).

**Table 1.** A comparison of direct assessment of practical skills, and indirect assessment of practical skills (Reiss et al., 2012, p. 7).

	DAPS	IAPS
**What is the principle of the assessment?**	A student’s competency at the manipulation of real objects is directly determined as they manifest a particular skill	A student’s competency at the manipulation of real objects is inferred from their data and/or reports of the practical work they undertook
**How is the assessment undertaken?**	Observations of students as they undertake a piece of practical work	Marking of student reports written immediately after they undertook a piece of practical work or marking of a written examination paper subsequently taken by students
**Advantages**	High validity Encourages teachers to ensure that students gain expertise at the practical skills that will be assessed	More straightforward for those who are undertaking the assessment
**Disadvantages**	More costly Requires teachers or others to be trained to undertake the assessment Has greater moderation requirements	Lower validity Less likely to raise students’ level of practical skills

In terms of potential implications for assessment of practical science in secondary school science, there are various aspects of innovation that OSCEs can offer. First, the OCSEs rely on a realistic case study for problem solving. A science lesson example could potentially include realistic problems for students to solve, such as disposal of unwanted chemicals in their schools. Second, the OCSEs present an opportunity to integrate medical knowledge with skill sets that need to complement the use of such knowledge. These include communication and professional skills. Without the integration of such skills, the assessment of practical science skills will be fairly limited. If we consider the example of the disposal of unwanted chemicals in schools, students can be expected to engage with their class as well as the wider school membership with constructive discussions about how best to manage the wellbeing of the community. Many science curriculum standards around the world, including the influential Next Generation Science Standards in the USA (NGSS Lead States, 2013) include reference to the importance of situating science in its societal context within science education. Coordination of various curriculum standards with the content of assessments will ensure harmony and cohesion within the education sector (Duschl, 2008).

The review of example innovations in assessment is intended to highlight what some features of innovation that could potentially be included in science assessments. The components of innovation are summarised in Table 2. A common feature of these assessments is that they target the measurement of the ability to engage in practical science in the context of complex problem-solving, collaborative engagement and inter/trans-disciplinary settings. These components can potentially be used as (a) indicators of quality in existing assessment documents, and (b) guiding features to inform design of new assessments.

**Table 2.** Example innovative assessments.

Assessment	Innovation
**PISA computer-based assessments**	Large-scale computerised assessment Collaborative problem-solving Use of avatars in assessment Digital literacy in reading scale
**Scenario analysis**	Investigate complex problems Trans-disciplinary knowledge and inquiry
**OSCEs**	Rotation around a number of stations to use information provided and analyse a given problem to reach a solution

Following on from a broad overview of literature background on high-stakes assessment of practical science, and subsequently focusing in on some example innovative approaches in assessments, we now turn to an empirical investigation of some selected set of summative assessment documents to address the guiding research question on the nature of assessment frameworks in relation to practical science.

## Methodology

### Research question

The overall question guiding the research is ‘What is the nature of assessment frameworks in relation to practical science in a sample of English-speaking countries that performed higher than the OECD average in the 2015 PISA science test?’ The main motivation underlying the question is the need to understand if there are aspects of high stakes summative assessments in these countries that can provide insight into how best to potentially structure national examinations so that they can mediate meaningful engagement in practical science in science lessons. The reference to ‘English-speaking’ is a pragmatic issue primarily due to the limitations of the authors in accessing documents published in other languages.

### Data sources

In order to address the main question, we performed a qualitative analysis and analysed the science summative assessments of a number of English-speaking countries. The main inclusion criterion focused on the country performing well in PISA science 2015 (i.e. national mean score in science is higher than the OECD average). PISA outcomes are often used as indicators of quality of education (REF). Tables of country rankings on PISA scores are often not only by educational researchers but also policy makers and politicians (Breakspear, 2012; Kjærnsli & Lie, 2011; Sellar & Lingard, 2014). However, there is widespread criticism about the content and processes involved in PISA (Sjøberg, 2019). For example, PISA rankings create panic and discomfort in practically all countries, including high-scoring countries (Alexander, 2012). Some researchers have highlighted some problematic aspects of measurement involved in PISA (e.g. Goldstein, 2017; Hopfenbeck et al., 2018; Kreiner & Christensen, 2014).

While we acknowledge such criticisms of PISA, we believe that for the purposes of the study, the PISA rankings can be used as a common benchmark on which to base the selection of countries in order to investigate their assessment frameworks. Considering the vast number of variables that exist among different countries’ assessment policies let alone the education systems, it is difficult to find a neutral benchmark to define the country selection criteria. PISA rankings exist and many countries’ national assessment bodies engage in them extensively. The PISA data present a basis on real school systems’ framing of high stakes assessments. In this sense, they are realistic and pragmatic, offering researchers a chance to compare their findings with other trends from data if needed.

### Data analysis approaches

A qualitative analysis was carried out to investigate particular summative assessment aspects including (a) construct of practical science, (b) format of assessment, (c) marking procedures and (d) mechanisms of quality control. The selection of countries was based on performance on the latest PISA science assessments where science was the assessed domain (OECD, 2018). Those countries with scores above the OECD average score were selected, and the following criteria were applied to refine the selection:

- English-speaking
- No restrictions in accessing the assessment material

Science assessments were targetted to be high stakes at age 16 (or equivalent in secondary education depending on the countries). This is because in our own context of England, this is the age for high stakes assessments and we were interested in comparing our own national context to other countries' assessment provisions. The definition of ‘high stakes assessments’ endorsed by the National Council on Measurement in Education (NCME) was adopted. The definition is as follows: ‘Tests whose results have important, direct consequences for the individuals, programs, or institutions involved’ (AERA, APA & NCME, 2014, p. 219). This definition suggests a testing programme for which there are important consequences (e.g. promotion, salary level, job retention, and required school reorganisation are examples) for students, teachers, administrators, or schools, based on the level of scores students attain.

- Science assessments include a practical science component (that is an opportunity for students to be involved in manipulating and/or observing objects and materials in order to demonstrate science understanding).

Applying the inclusion criterion of ‘high stakes at age 16’ led to the elimination of Australia (e.g. New South Wales and Queensland was reviewed) and Ireland from the list. The focus on the age group was guided by two factors: (a) our national context where high stakes assessments take place at age 16. We wanted to explore other country’s assessment provisions so as to determine if there are useful lessons to be learned for our own context; and (b) consistently top performing country (e.g. Singapore) have a high stakes examination at age 16. For a fair comparison in terms of the student age at high performing examination systems, the following English-speaking countries were chosen: Singapore, USA (Vermont and New York), Canada (Alberta), New Zealand and UK (England). For these countries, information was compiled to respond to the following questions:

- What is the construct of practical science that is being assessed?
- What is the format of the assessment? (e.g. teacher-assessment, e-assessment, written exam, portfolio, oral assessment)
- Who does the marking? How is the marking carried out?

These questions are implied by issues raised in the review of research literature in relation to the summative assessment of practical science. For example, the question about practical science being curriculum-based implicitly refers to the frequent misalignment between curriculum and assessment (e.g. Chudowsky & Pellegrino, 2003). The question ‘What is the construct of practical science that is being assessed?’ focuses on ‘practical science’ as an important definition to tease out in the documents even if a definition may not be an explicit feature of the document itself. Furthermore, key issues of reliability and validity of assessments are addressed through the questions about how the marking is carried out. The central issues in design of summative assessments, such as cheating, format of assessments and reliability of markers (e.g. Ercikan & Barclay-McKeown, 2007) are thus taken into account.

Collectively, the questions provide analytical categories for tracing the content of the assessment documents. In other words, they are inclusive of themes that are taken to be important features for analysis. In this sense, the questions are laden with theoretical assumptions and themes that are guiding our analysis. Such themes differentiate our approach from simply being a descriptive account of what the content of the documents is about. A descriptive account would simply present the content without an analytical lens to delineate components of the documents. The data analysis approach, then, involves a considerable level of thematic interpretation which is used to compare and contrast the different countries’ assessment frameworks.

Overall, the questions provide a comprehensive account that addresses concerns about summative assessment of practical science for high stakes purposes. The assessment frameworks from Singapore, USA (Vermont), Canada (Alberta), New Zealand and UK (England) were investigated to answer the questions. Qualitative description of the responses to each question was noted in each case, and trends across the documents were investigated. The purpose in the analysis was not to be exhaustive about all English-speaking countries but rather it is to provide some examples about a range of aspects, such as the construct of practical science and contextual information about the assessment system (e.g. who does the marking and how?). Ultimately our aim is to trace some qualitative trends in how these countries situate practical science in their high-stakes assessments and what features of innovation might be available in different assessment systems.

## Results and findings

The key findings related to Singapore, USA (Vermont), Canada (Alberta), New Zealand and UK (England) are presented in this section. In each case, the outcomes of the qualitative document analysis are presented along with description of the national context for the assessment frameworks.

### Singapore

From 2018 onwards, skills of practical science are assessed in Singapore using a summative end-of-course assessment, supported by a teaching fraternity familiar with practical competencies after a decade of school-based practical assessment. Depending on pupils’ performance at the compulsory national primary school leaving examinations (PSLE) for 12-year olds, mainstream pupils enter three major pathways with end-of-course certifications known as Ordinary-level, Normal-Academic and Normal-Technical. O-level pupils undertake 4-years of secondary education while N-level pupils can take up to 5. The N-T pathway equip pupils for a predominantly post-secondary vocational education. Certification is jointly administered by Ministry of Education and Cambridge International Examinations (CIE). The key findings about the Singapore assessment system is provided in Table 3.

### USA

Considering the vast range of educational provision across the different states of the USA, it is beyond the scope of this paper to review the assessment systems of all states. Decentralised education system, states, districts and schools can, to varying degrees, introduce and implement their own standards. As an example, in New York all high school students are required to complete three science courses, including a science course that incorporates 1200 min of laboratory activity in order to graduate (Champagne & Shiland, 2004). To assess laboratory learning in these science courses, the state has for many years administered an examination in each subject consisting of both a written test with tasks related to laboratories (such as items asking about laboratory techniques and design of experiments) and a laboratory performance test. The planned new Physical Setting/Earth Science Performance Test included hands-on tasks to be completed at six stations in a secure laboratory classroom. Students are be tested on their ability to identify minerals, locate an earthquake epicentre, measure atmospheric moisture, determine the density of different fluids, collect and analyse data on the settling of particles in a column of fluid, and construct and analyse an elliptical orbit (DeMauro, 2002).

Currently, 20 states have adopted the *Next Generation Science Standards*, affecting 35% of the US pupil population (OECD, 2017). An additional 25 states have adopted the Framework for K-12 Science Education (National Research Council, 2012). Here we focus on one state, Vermont which has adopted NGSS. Considering the importance attributed to NGSS in recent trends in science education (Lin et al., 2019), it may be informative to investigate how an example American state, Vermont, is adjusting its assessment provision in light of NGSS. In Vermont, the NGSS-aligned assessment is administered to students in grades 5, 8, and 11 unless a student qualifies for alternate assessment or an exemption for medical reasons. This represents a change from practice in 2016–2017, when the New England Common Assessment Program (NECAP) assessment was administered to grades 4, 8, and 11.

The new test administered via computer is intended to use of innovative item clusters that will make it possible to measure the full breadth of the NGSS standards. The assessment includes reports to parents and schools that clearly articulate student performance. Additionally, the assessment includes accommodations and accessibility features to provide access for a broad range of diverse student needs in Vermont. The NECAP assessment assesses an inquiry task on paper. Vermont used an inquiry task on paper which measures the ability to think scientifically. The task requires test-takers to hypothesise, plan and critique investigations, analyse data, and develop explanations. Vermont has announced that future inquiry tasks will be aligned to the NGSS standards. A summary of the key findings from Vermont, USA are provided in Table 4.

### Canada (Alberta)

Canada’s education system is devolved with every provincial jurisdiction being responsible for its own education provision. We examined specifically how practical science is assessed at age 15–16 in three populous provinces: Alberta, Ontario and Quebec. While practical science is one of the major goals of the science curriculum in Ontario, the skills are not assessed in a high-stakes environment.^1^ Evidence of student achievement for evaluation is collected over time from three different sources – observations, conversations, and student products. In other parts of Canada, such as Quebec, 15/16-year – old students take the high-stakes Epreuves Uniques in their 4th year of secondary school. The science curriculum highly focuses on practical science. The mark on the test accounts for 50% of the final score. Students must have a passing mark (normally 60%) to obtain the Diplôme d’Etudes Secondaires (i.e. school exit examinations). Unfortunately, access to exemplar tasks was not possible.

In Alberta, Grade 9 students typically sit the Provincial Achievement Tests (PATs) at the end of the school year.^2^ PATs consist of multiple – choice questions which could sometimes assess practical science skills. The purpose of the PATs is to:

- Determine if students are learning what they are expected to learn
- Evaluate how well students have achieved provincial standards at given points in their schooling
- Assist schools, authorities, and the province in monitoring and improving student learning

Findings from Alberta assessment documents are summarised in Table 5.

### New Zealand

The National Certificate of Educational Achievement (NCEA) is the official secondary school qualification in New Zealand. NCEA consists of a combination of internal and external assessments. Internal assessments are used to assess skills and knowledge that cannot be tested in an exam, e.g. speeches, research projects and performances, such as those require for practical work in science. Practical activities do not provide students with a complete set of instructions to follow. Instead, students have some freedom to adopt procedures they choose and decide how to record, analyse and report the data collected.

The National Certificate of Educational Achievement (NCEA) is the official secondary school qualification in New Zealand. NCEA has three levels, one for each of the last three years of secondary school (age 15-18). NCEA consists of a combination of internal and external assessments. The NCEA is said to be the only secondary school qualification worldwide where marked examination papers are returned to students. There are no limitations on the schools from offering only internals/ or externals. Internal assessments are set and marked by teachers, with grades checked by other teachers and samples in turn checked by the New Zealand Qualifications Authority (NZQA). That process and the overall integrity of NCEA is overseen by an independent advisory group, the Technical Overview Group Assessment (Singh, 2014).

Students are assessed both internally and externally in the New Zealand system. The NCEA level one that occurs in Year 11 is the equivalent to GCSE level. To gain a level certificate with Merit endorsement, a student must pass the level with at least 50 Merit and Excellence credits assessed at that level or higher. Internal assessments are used to assess skills and knowledge that cannot be tested in an exam, e.g. speeches, research projects and performances, such as those require for Practical work in science. The teachers are provided with example internal assessment resources from the NCEA where it is urged that they are adapted by the teacher. It states that the resources are guides for effective assessment and should not be used as actual assessments as they are publicly available. In order to ensure authenticity and demonstrate that the student is capable of applying what they know, teachers are required to modify the context, topic or figures used in the supplied resource investigations. Each practical is worth four credits and the student’s time to complete the work varies depending on the project.

The internal assessment is administered in schools by teachers. However, the assessment tasks pre-moderated by the New Zealand Qualifications Authority (NZQA) and assessments are moderated externally on a three-year cycle basis. For this the school selects randomly three to four assessments that have been given each assessment category; Not achieved, Achieved, Merit or Excellence and sends them to NZQA. The grade does not change for the students, but the school and teachers receive feedback on their marking. Internally in most science departments, assessments are moderated across the department. If there is only one teacher, then they get a colleague from a neighbouring school to moderate for them. Schools can choose to offer the practical investigation for internal or choose amongst other internal assessments which are ‘research’ based. Procedure for both is the same. Practical activities do not provide students with a complete set of instructions to follow. Instead, students have some freedom to adopt procedures they choose and decide how to record, analyse and report the data collected. The overall findings are summarised in Table 6.

### UK (England)

The United Kingdom consists of England, Wales, Scotland and Northern Ireland. Similar to the cases of USA and Canada, it is beyond the scope of this paper to review the assessment provision in all nations in the UK. We have focused on England because it is our own national context. Understanding this context will help us in our subsequent research and development on summative assessment of practical science. The education system in England underwent a set of reforms beginning in 2010 that resulted in a revised curriculum for many subjects to be taught from 2015 onwards (Childs & Baird, 2020). Importantly these reforms also brought in a new assessment regime beginning with some core subjects in 2017, with the other subjects, including the sciences, following in 2018–2019. As part of these reforms, non-exam assessments such as coursework or controlled assessments were removed from all subjects other than those where a student’s performance (e.g. dance or drama) or the production of physical objects (e.g. art) are the most valid expression of their subject skills and competencies. As a consequence, the vast majority of post-reform subjects are now assessed at age 16 (GCSEs) or 18 (A-levels) using only a set of written exam papers.

GCSE stands for ‘General Certificate of Secondary Education’ and it is an academic qualification generally taken in a number of subjects by pupils in secondary education in England, Wales and Northern Ireland. Each GCSE qualification is in a particular subject such as biology, physics and chemistry and stands alone (although a set can also be pursued). Studies for GCSE examinations generally take place over two or three years depending on the subject. Science subjects no longer include a ‘hands-on’ assessment of practical science skills as they did pre-reform; instead, the final exam papers are intended to have items specifically written to indirectly assess students’ knowledge and understanding of practical science (Ofqual, 2015).

In the current assessment landscape in England, the assessment of practical science skills has been contentious (Childs & Baird, 2020). Science subjects including chemistry no longer include a ‘hands-on’ assessment of practical science skills. Instead, the final examination papers are intended to have items specifically written to indirectly assess students’ knowledge and understanding of practical science (Ofqual, 2015) The Office of Qualifications and Examinations Regulation advocates that at GCSE level (ages 14–16) pupils should ‘develop their ability to evaluate claims based on science through critical analysis of the methodology, evidence and conclusions, both qualitatively and quantitatively’ (Ofqual, 2015, p. 5). Ofqual is a non-ministerial government organisation that regulates qualifications, exams and tests in England and, until May 2016, vocational qualifications in Northern Ireland.

Overall, our analysis indicates that countries and jurisdictions that kept high-stakes examinations at age 16 adopted different formats of assessments of practical science from teacher-based internal assessments to external assessments. In England, assessment of practical science has been exclusively in a written format. A similar approach seem to be the case for Vermont, USA although there is more of a narrative approach to the reporting of practical science in the assessments. Across all the countries, the construct of practical science involves elements of the planning, implementation and evaluation of practical aspects of science, although it is not entirely clear how the skills of implementation of practical science is written assessments.

In locating the data from different countries, we noted that it was not always possible to access the actual examination questions. Hence, we could not carry out an analysis of actual assessment tasks for all the countries involved. Here we provide two examples, one from Alberta, Canada (Appendix 1) and another from New Zealand (Appendix 2). These examples provide illustration of different approaches to assessment including multiple choice written items (in the case of Alberta, Canada) and hands-on practical skills (in the case of New Zealand). As Appendix 2 shows, there is also a fairly thorough rubric to assess the performance around the practical investigation. The appendices can be accessed at https://doi.org/10.1080/09500693.2020.1769876.

Apart from New Zealand (which seems to have a more interdisciplinary and holistic approach to assessment) and in particular assessment of practical science, the other countries reviewed do not seem to have adopted innovative ways of assessing practical science. By innovative, we meant types of tasks that are different from the common pen-and-paper assessments that target knowledge of practical science (e.g. naming a product in a chemical reaction or describing certain procedures). Some examples of innovative assessments were reviewed in Table 2. For instance, the use of computerised assessments on a large scale (exemplified by PISA tasks), integration of transdisciplinary knowledge and inquiry (e.g. scenario analysis) and interactive problem solving (e.g. OSCEs) would constitute as indicators of innovation in assessment. While teacher assessments may be considered innovative depending on their nature, the idea of teacher assessment is not entirely new, having been utilised on a national scale for high-stakes testing in different countries, for example in England (Ofqual, 2013).

It should be noted that a shortcoming of our investigation into the assessment frameworks is that given the diversity of source and content of the different countries’ jurisdictions, it was not possible to generate a consistent set of themes across the questions being investigated. This is because the information was more often than not partial or presented in vastly different ways across the countries. For example the data reported in the Tables 3–7 were summaries of information to address the questions but they originated from very different parts of the actual documents or websites that are available in each country. The data sources do not have a uniform organisational structure nor do they fit a uniform set of themes that are readily identifiable. Despite such limitations, we believe that the outcome highlights some indicative information on what some high performing OECD countries are assessing about practical science and how. For instance, the overreliance of written assessments in England is informative. Likewise the fact that all countries reviewed have ‘evaluation of experimental data’ as a common aspect of practical science is interesting. Future research can focus on in-depth investigations into each country case, and involve more rigorous thematic analysis (Nowell et al., 2017) to establish finer grain similarities and differences in assessment policies.

**Table 3.** Findings from Singapore high stakes assessments.

Guiding questions	Observations
Is this a high-stakes test at age 16?	Science is high-stakes at age 16 for pupils attempting O-level and N-A certification.
Is practical science assessed?	Only for O-level pathways. N-level pathways have no practical assessments.
What is the format of the assessment?	End-of-course practical examination in O-levels which is taken in a single sitting.
What is the construct of practical science that is being assessed?	Experimental skills and strategies Follow a sequence of instructions Use techniques, apparatus and materials Make and record observations, measurements and estimates Plan investigations, select techniques, apparatus and materials Analysis and evaluation Interpret and evaluate observations and experimental results Evaluate methods and suggest possible improvements. 4 skill areas identified Planning (*P*) Manipulation, measurement and observation (MMO) Presentation of data and observations (PDO) Analysis, conclusions and evaluation (ACE) One or more of the questions may incorporate assessment of planning and require candidates to apply and integrate knowledge and understanding from different sections of the syllabus.
Who does the marking? How is the marking carried out?	Scripts send to Cambridge International Examinations and marked by UK examiners.

**Table 4.** Findings from Vermont, USA high stakes assessments.

Guiding questions	Observations
Is this a high-stakes test at age 16?	Yes. Driving forces influencing high-stakes component of science courses are (a) state high school graduation requirements; and (b) state requirements for higher education admissions (e.g. college and university entrance requirements). More than half of the US states required pupils to complete at least 2 years of high school science.
Is practical science assessed?	Yes. There are some laboratory-based science course completion requirement before they can graduate. Many of these assessments are selected response tasks, scored by computer. There are very few have hands-on performance assessments, due to reliability and pragmatic reasons.
What is the format of the assessment?	The format varies. From computer administered, or written MCQ and open-ended questions based on required and identified lab activities to totally hands-on investigations in single sitting.
What is the construct of practical science that is being assessed?	The assessment task requires test-takers to hypothesise, plan and critique investigations, analyse data, and develop explanations. According to the administrative instructions, pupils read a short story, and then make predictions based on the information in the story. Next, they answer questions related to the story. Vermont has announced that future inquiry tasks will be aligned to the NGSS standards
Who does the marking? How is the marking carried out?	Trained scorers or teachers.

**Table 5.** Findings from Alberta, Canada high stakes assessments.

Guiding questions	Observations
Is this a high-stakes test at age 16?	Yes.
Is practical science assessed?	Yes. For example, there is reference prediction and a hypothesis based on background information or observations.
What is the construct of practical science that is being assessed?	The assessment framework focuses on a broad-based skills approach. These skills are identified as (a) identify and analyse problems; (b) explore and test solutions; and (c) seek, interpret and evaluate information.
How is the marking carried out?	The Grade 9 Science Achievement Test consists of 55 machine-scored questions: 50 multiple-choice questions, each worth one mark, and five numerical-response questions, each worth one mark. The five numerical-response questions are interspersed among the multiple-choice questions.

**Table 6.** Findings from New Zealand high stakes assessments.

Guiding questions	Observations
Is this a high-stakes test at age 16?	Yes. To gain a level certificate with Merit endorsement, a student must pass the level with at least 50 Merit and Excellence credits assessed at that level or higher.
Is practical science assessed?	Yes. There is internal assessment by the teacher. At Level 1, there are 27 suggested internal assessment resources for practical work, but teachers are instructed to change these with different numbers and figures where appropriate.
What is the format of the assessment?	Combination between internal and external assessments. Pupils write a report on their work. Either this is two practical classes to gather data and then two other classes to analysis and write up on the data. This is continuous over the one-year programme. And is done for the subsequent exam years.
What is the construct of practical science that is being assessed?	School science investigations are construed to be a set of instructions to follow but have some freedom to choose the procedures to follow, and to decide how to record, analyse and report the data collected. They may also (though this will not be taken as a defining characteristic) have some freedom to choose the question to be addressed and/or the final conclusion to be drawn.
How is the marking carried out?	Teacher assessed. This is internally in most science departments and it is moderated across the science department. If there is only one teacher in a particular school, then they get a colleague from a neighbouring school to moderate for them. Schools can choose to offer the practical investigation for internal or choose amongst other internal assessments which are ‘research’ based. Procedure for both is the same.

**Table 7.** Findings from England, UK high stakes assessments.

Guiding questions	Observations
Is there a high-stakes test at age 16?	Yes. The tests are referred to as ‘General Certificate of Secondary Education’ (GCSE). GCSE is an academic qualification generally taken in a number of subjects by pupils in secondary education in England, Wales and Northern Ireland
Is practical science assessed?	Yes. The Office of Qualifications and Examinations Regulation advocates that at GCSE level (ages 14-16) pupils should ‘develop their ability to evaluate claims based on science through critical analysis of the methodology, evidence and conclusions, both qualitatively and quantitatively’ (p. 5).
What is the format of the assessment?	From September 2016 practical skills in the new GCSE are assessed by written examination only. The examinations require students to demonstrate their understanding of scientific experimentation, with 15% of the total marks being allocated to each science GCSE.
What is the construct of practical science that is being assessed?	The *Working Scientifically* strand is the primary source that refers to the construct of practical science. The strand consists of (a) the development of scientific thinking, (b) experimental skills and strategies, (c) analysis and evaluation and (d) vocabulary, units, symbols and nomenclature
Who does the marking? How is the marking carried out?	There are three main examination boards: AQA, EdExcel and OCR. AQA and Edexcel, there are eight ‘required’ or ‘core’ practicals for each single GCSE in biology, chemistry and physics. OCR took a slightly different approach in each subject by identifying Practical Activity Groups (PAGs) and then giving examples of practicals that are suitable for the particular PAG. Examiners from the exam boards carry out the marking.

## Conclusions and Discussion

In this paper, we examined science assessments of countries demonstrating high performance in PISA science assessments in order to understand if there is any innovation in summative assessments of practical science. The review was restricted to countries which assess science at age 16 in a high stakes fashion. The findings illustrate that the examined countries’ jurisdictions used different approaches from traditional external pen-and-paper tests to internal teacher assessments that included different formats (e.g. report). The types of innovations that are embedded in examples such as PISA computerised tasks, scenario analysis and OSCEs were not part of these high-stakes assessments.

A further outcome of the qualitative analysis was the identification of the construct of ‘practical science’ being assessed. The construct ranged between the countries whose assessments were examined. For example, whereas Alberta, Canada assessments included learning outcomes such as teamwork, New Zealand assessments were based on fairly open-ended investigations. Singapore assessments consisted of fairly focused references to aspects of practical science such as ‘manipulation, measurement and observation; presentation of data and observations; and analysis, conclusions and evaluation’. However, overall there were similar concepts about practical science in terms of how students should learn about the planning, implementation and evaluation of practical science.

Research evidence suggests that external examinations not closely tied to curriculum and instruction do not provide valid evidence of student learning (Linn et al., 1991). The alignment of assessment with curricular goals and teaching approaches is critical to supporting learning. Based on research that promotes links between learning and large – scale assessments (Chudowsky & Pellegrino, 2003; Ercikan & Pellegrino, 2017) some key assessment design elements have been identified. Ercikan and Barclay-McKeown (2007) point out that first and foremost requirement is the clarity about the underlying constructs to be assessed. One of the challenges in developing assessments that take into account the development of knowledge and competence is the limited amount of knowledge about learning in particular content areas, and how progression of learning can be linked to performance on assessment tasks.

The question remains about the mode and format of innovative high-stakes assessments and whether pen-and-paper assessments can validly assess more complex accounts of the construct of practical science. In addition, it is worth considering whether or not the low level of innovations in these assessments is due to lack of creativity or whether innovations conflict with many other considerations including pragmatic ones that are associated with the high-stakes nature of the summative assessments. The design of future assessments for high stakes examinations will benefit from clear articulation of the construct of practical science and how the construct can be tested reliably on a large scale. Although the example innovations such as computerised PISA tests and scenario analysis are promising, they are not yet part of national assessment frameworks and further research is warranted in exploring if and how they can be adopted for high stakes purposes.

## Supplementary Material

Supplemental Material

## Notes

1 Ontario Canada Science curriculum: http://www.edu.gov.on.ca/eng/document/curricul/secondary/oss/epp.pdf

2 Note that Grade 6 students in Alberta also sit PATs.
