# Supplementary figures and images for: Assessment of practical science in high stakes examinations: a qualitative analysis of high performing English-speaking countries
Source: Int J Sci Educ. 2020 Aug 6;42(9):1544–67. doi: 10.1080/09500693.2020.1769876 (PMC7872216; doi:10.1080/09500693.2020.1769876)

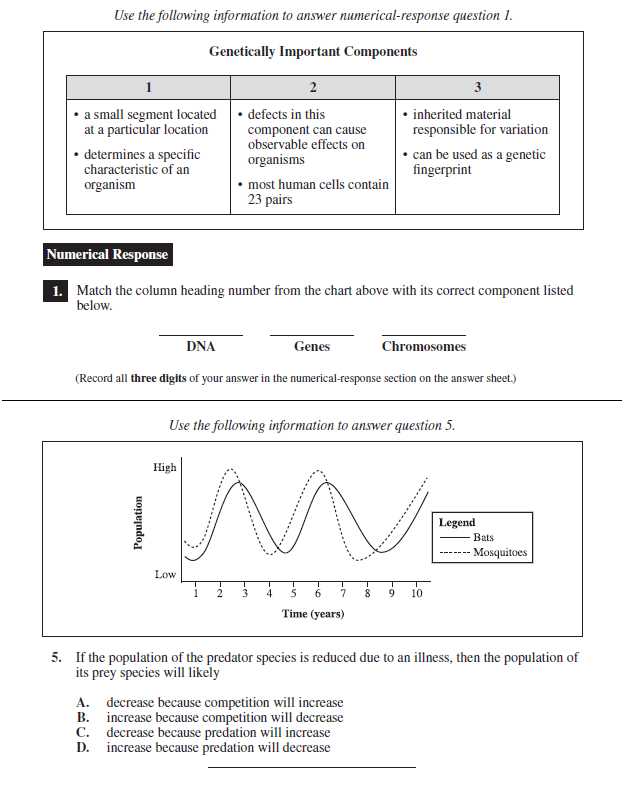

Supplement: Supplemental Material [file TSED_A_1769876_SM1434.zip › SUPPLEMENTAL FILES/APPENDIX_1_tsed_2019_0629_a_File006.docx]

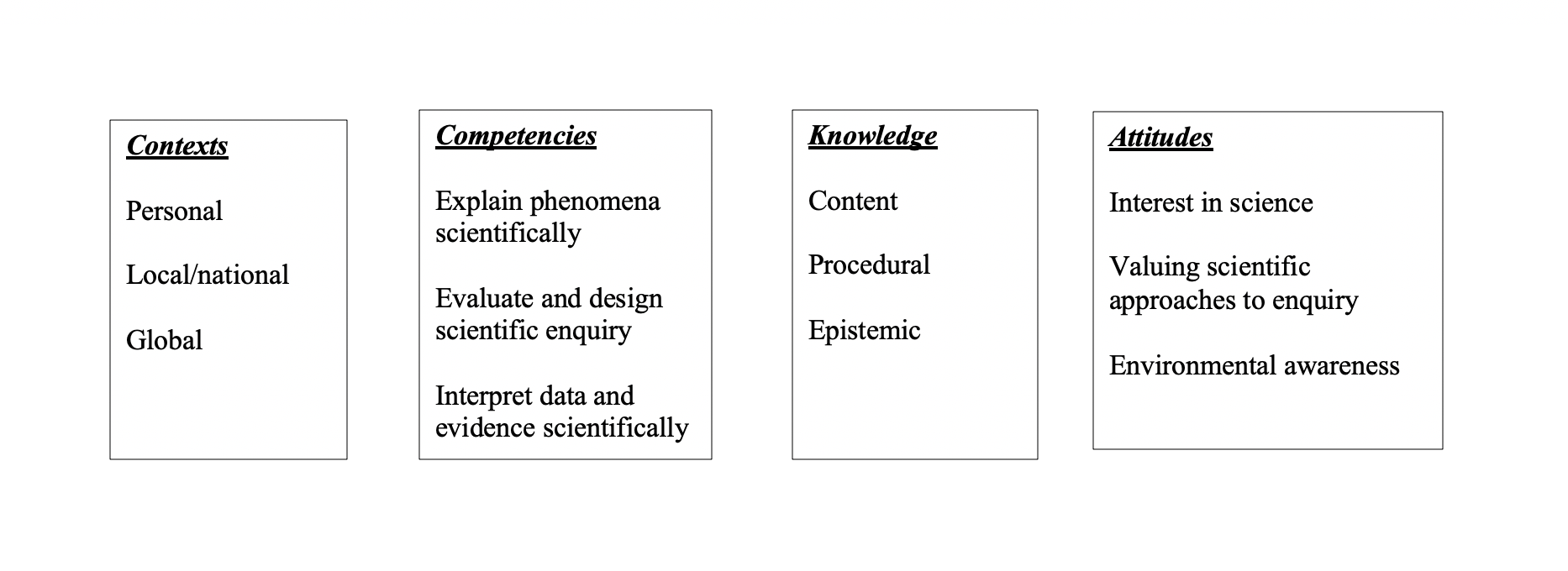

Supplement: Supplemental Material [file TSED_A_1769876_SM1434.zip › SUPPLEMENTAL FILES/Figure_1.tiff]

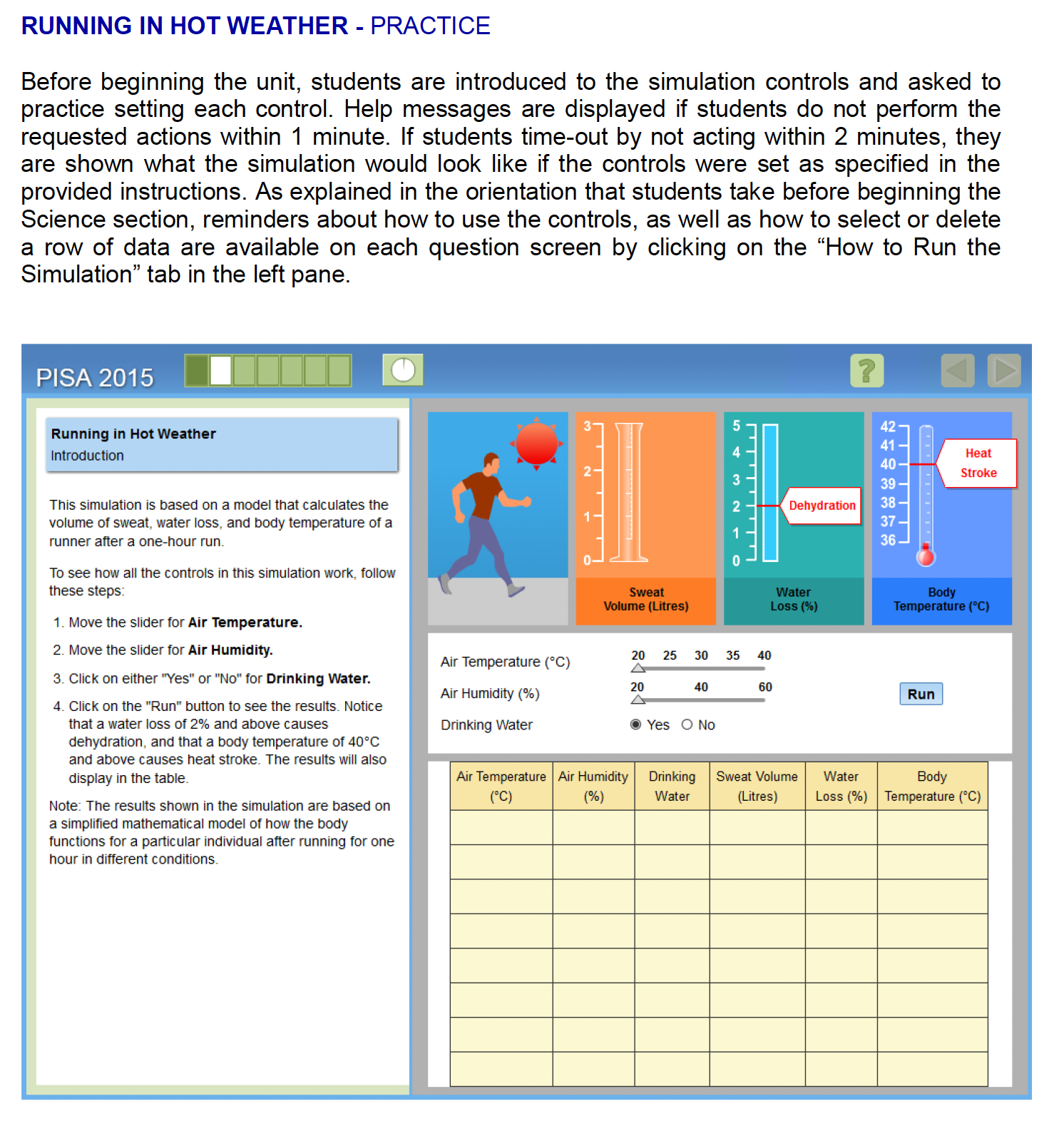

Supplement: Supplemental Material [file TSED_A_1769876_SM1434.zip › SUPPLEMENTAL FILES/Figure_3.jpg]
