# Supplementary material for: Assessment of practical science in high stakes examinations: a qualitative analysis of high performing English-speaking countries
Source: Int J Sci Educ. 2020 Aug 6;42(9):1544–67. doi: 10.1080/09500693.2020.1769876 (PMC7872216; doi:10.1080/09500693.2020.1769876)
Supplement: Supplemental Material [file TSED_A_1769876_SM1434.zip › SUPPLEMENTAL FILES/APPENDIX_2_tsed_2019_0629_a_File007.doc]

Achievement Standard Science 90943: Investigate implications of heat for everyday life

Resource reference: Science 1.4A v3

Resource title: Home Insulation

Credits: 4

| Achievement | Achievement with Merit | Achievement with Excellence |
| --- | --- | --- |
| Investigate implications of heat for everyday life. | Investigate, in depth, implications of heat for everyday life. | Investigate, comprehensively, implications of heat for everyday life. |

Student instructions

Introduction

Many homes in New Zealand have insufficient insulation. People who live in poorly insulated homes must either live in a cold, damp home or use large amounts of energy for heating. Reducing heat losses from homes is therefore of considerable public importance. Two methods of reducing heat loss are to use fibreglass pads or polystyrene sheets under floorboards, in wall cavities, or in ceiling spaces.

This assessment task requires you to find out and explain whether fibreglass is a better insulator than polystyrene for home insulation, and why.

You will gather data in experiments you conduct in groups of up to three students. You will independently conduct further research.

You will independently use the information you collect and your experimental results to prepare a report that describes your findings and explains the impact of this aspect of heat on everyday life.

You have one week of in-class and out-of-class time to complete this work.

Teacher note: Adapt the time allowance to suit your context. Specify a due date, e.g. “Your due date is…”

You will be assessed on how well you are able to research the insulation properties of the two materials and draw conclusions that are linked to physics theory about this aspect of heat and its implications for everyday life.

You will not be assessed on how you gather your data.

Task

See Student Resource A for further guidance.

Gather primary data

Your teacher will supply you with suitable equipment and material that will enable you to measure the insulation properties of fibreglass and polystyrene across the temperature range of 0º to 40º Celsius.

Teacher note: Provide each group with appropriate apparatus to produce a temperature gradient in the range of 0º to 40º Celsius, e.g. beakers, a thermometer and retort stand, and pieces of fibreglass and polystyrene insulation, such as Batts and Expol.

In a group of up to three students**,** set up and carry out an experiment that tests the ability of the two different materials to prevent heat loss in a typical New Zealand home.

Teacher note: Provide specific guidance or instructions to students about how to carry out the experiment(s).

Make sure each member of your group agrees on and records the same data.

Gather secondary data

Working independently, collect relevant information on insulation from a range of sources.

Collect enough information to allow you to discuss the links between heat, home insulation, scientific theory, and the results of your experiment.

Teacher note: You could provide or adapt the planning template in Teacher Resource A.

Prepare and present your report

Working independently, write a report that explains the impact of heat loss and insulation on the everyday life context of home insulation.

Include your data with your report.

Student Resource A: Further guidance

Gathering data

Your data should allow you to determine:

- for the same thickness of insulation material, which is the best insulator
- what thickness of fibreglass is equivalent to one polystyrene section
- how fibreglass and polystyrene compare to a control insulator, for example an air gap.

Record your data in an organised way. For example, you could draw diagrams showing different configurations of your apparatus, and the corresponding results.

Preparing and presenting your report

In your report:

- discuss how the insulation ability of each material compares with each other
- give a scientific description of how the physical properties of materials affect their ability to insulate (prevent heat loss)
- link the data you have gathered to scientific theory, for example by providing scientific reasons why one material was a more efficient insulator than the other
- describe how heat is lost in a typical home
- explain why heat loss should be prevented.

Use scientific statements, show calculations, and state units in your report, as appropriate.

Show evidence of your own critical thinking. For example, you might elaborate, justify, relate, evaluate, compare and contrast, or analyse the science information you have gathered.

Assessment schedule: Science 90943 Home Insulation

| Evidence/Judgements for Achievement | Evidence/Judgements for Achievement with Merit | Evidence/Judgements for Achievement with Excellence |
| --- | --- | --- |
| The student investigates implications of heat for everyday life.  The student:   - gathers and processes data about the relative insulation properties of fibreglass and polystyrene insulation material, and conducts further research into their properties - describes how science is involved in home insulation - provides in their report a general description of how heat is lost (heat transfer mechanisms), and may include a diagram or narrative description of experimental results - describes link(s) between the physics theory of heat transfer, heat loss in homes, and the two types of insulation material.   For example (partial), the student describes most of the methods of heat transfer and the effects of insulation:  Heat flows from a hotter region to a colder region.  Heat transfer in this context will predominantly be via conduction.  Having a poor conductor between the two regions reduces conduction, and radiation or convection.  Insulation under the floorboards or in other areas will also reduce winds or draughts into the house. | The student investigates, in depth, implications of heat for everyday life.  The student:   - gathers and processes data about the relative insulation properties of fibreglass and polystyrene insulation material, and conducts further research into their properties - explains how science is involved in home insulation - provides in their report an explanation of how heat is lost (heat transfer mechanisms), and may include a diagram or narrative description of their experimental results - explains link(s) between the physics theory of heat transfer, heat loss in homes, and the two types of insulation material.   For example (partial), the student explains most of the methods of heat transfer and the effects of insulation:  Heat flows from a hotter region to a colder region.  Heat transfer in this context will predominantly be via conduction.  Having a poor conductor between the two regions reduces conduction, and radiation or convection.  Insulation under the floorboards or in other areas will also reduce winds or draughts into the house.  The student’s report also includes several of:  Report describes the structure of the materials in relation to their ability to be a poor conductor, related to the small air pockets available.  Commercial R-values and how they relate the experimental data.  Explanation of why the structure of the materials reduces convection.  Recommendations about how the installation of the insulating materials should be performed based on the evidence from their data.  Estimate of appropriate floor/wall/ceiling thickness of insulation, based on experimental data. | The student investigates, comprehensively, implications of heat for everyday life.  The student:   - gathers and processes data about the relative insulation properties of fibreglass and polystyrene insulation material, and conducts further research into their properties - provides reasons and links these together to clearly explain how science is involved in home insulation - provides in their report a discussion of how heat is lost (heat transfer mechanisms) including the key heat transfer methods, and may include a diagram or narrative description of their experimental results - explains key link(s) between the physics theory of heat transfer, heat loss in homes, and the two types of insulation material.   For example (partial), the student explains most of the key methods of heat transfer and the effects of insulation:  Heat flows from a hotter region to a colder region.  Heat transfer in this context will predominantly be via conduction.  Having a poor conductor between the two regions reduces conduction, and radiation or convection.  Insulation under the floorboards or in other areas will also reduce winds or draughts into the house.  The student’s report also includes several of:  Report describes the structure of the materials in relation to their ability to be a poor conductor, related to the small air pockets available.  Commercial R-values and how they relate the experimental data.  Explanation of why the structure of the materials reduces convection.  Recommendations about how the installation of the insulating materials should be performed based on the evidence from their data  Estimate of appropriate floor/wall/ceiling thickness of insulation, based on experimental data.  Examples of critical evaluation/examination include:  Experimental apparatus and data are scaled up to an estimate of actual performance of home insulation, to allow valid comparisons.  Consideration of the relative amounts of the three types of energy transfer and how significant each type is.  Discussion of optimum thicknesses and applications for each type of material.  Discussion of limitations of their experiment and effect on their conclusions.  Description of variations between their experimental data and any prior or researched science knowledge, and explanations for why these variations may have happened.  Science reasons why radiation and convection are not likely to be influential in answering the question.  Discussion of how each insulation type is affected by condensation or water absorption with science reasons why and how each type of material might be affected.  Discussion of whether there should be gaps above any or both of the insulation types.  Relative abilities of each type of insulation to retard fire or mitigate the effects of winds or air flow. |

Final grades will be decided using professional judgement based on a holistic examination of the evidence provided against the criteria in the Achievement Standard.
